# Pathway analysis of time of pacifier use by children whose mothers are hearing impaired or have normal hearing

**Published:** 2020-12-11

**Authors:** Larissa Carcavalli, Carolina Castro Martins, Raquel Fabiane Nogueira, Fernanda Ruffo Ortiz, Lucas Rodrigues Teles, Saul Martins Paiva, Júnia Maria Serra-Negra

**Affiliations:** Department of Pediatric Dentistry, Universidade Federal de Minas Gerais, Av. Antônio Carlos, 6627, Pampulha, Belo Horizonte, MG, Brazil

**Keywords:** anxiety, breastfeeding, deafness, epidemiology, pacifier

## Abstract

**Background:**

The lack of mothers’ understanding and information can increase their anxiety and lead to unhealthy behaviors, such as pacifier-use, in their children.

**Aim:**

This study aimed to perform pathway analysis of pacifier use by children whose mothers were hearing impaired or had normal hearing.

**Methods:**

A cross-sectional study was conducted with 116 Brazilian mothers (29 hearing impaired and 87 with normal hearing). Mothers were interviewed about socioeconomic factors and their children’s pacifier use habits. They also completed the deaf or hearing versions of the Brazilian Beck Anxiety Inventory. The pathway analysis was used to determine the effects of different variables in the two groups.

**Results:**

The child pacifier use pathway among hearing mothers was associated with a long duration of pacifier use (*P*=0.005), bottle-feeding use (*P*=0.004), and mothers who had maternity leave (*P*=0.004). The child pacifier use pathway among mothers who were deaf was associated with premature birth (*P*=0.025) and a short duration of pacifier use (*P*=0.005). Mothers who were deaf were also more anxious than those who have normal hearing (*P*=0.002).

**Conclusions:**

Children of normal hearing mothers used a pacifier for a longer duration than the children of mothers who were deaf. Time of pacifier use was also directly affected by bottle-feeding and maternity leave.

**Relevance for Patients:**

These findings provide important information about the cultural path of pacifier use taken by families that had members with and without hearing impairment.

## 1. Introduction

There are nearly 360 million people worldwide with hearing impairment and they may experience problems with gaining access to healthcare facilities due to communication barriers [1]. Healthcare providers are trained for verbal communication and may not be able to communicate effectively with individuals with hearing impairment (this applies to both children and older adults), jeopardizing their access to health services [2].

The relationship between a mother and child is very important, as the mother is the main caregiver who influences the habits that are established in her children [3]. Anxiety may arise among mothers with hearing impairment because they are aware that their limited communication skills may jeopardize communication with healthcare practitioners regarding their children’s health [4,5]. Women with hearing impairment also report an incomplete understanding of the information shared by healthcare providers during prenatal care [5]. This lack of understanding can increase anxiety among mothers with hearing impairment and can also cause unhealthy behaviors when taking care of their newborns [4]. Moreover, normal-hearing mothers can become anxious when they hear their babies cry and may feel that they need to stop the crying [4].

Communication in response to a child’s crying clearly differs between the mother who is deaf when compared with the reaction of a mother with normal hearing [6-8]. Mothers can use a pacifier to calm a baby and prevent crying [9]. If a mother hears a baby crying, she may feel anxious to stop the crying, but if a mother who is deaf is unable to hear the crying, she may not feel any discomfort [6,7,9]. For example, if a mother hears her baby crying, she may feel anxious and want to stop the crying, so she would use a pacifier to calm the baby and prevent crying [9]. Whereas, a mother who is deaf and unable to hear her baby crying might not feel any discomfort [6,7]. Mothers with heating-impairment use their other senses, such as sight and touch, to communicate with their children.

The presence of one or more people with hearing impairment in a family leads to specific language and communication behaviors. Families with a person who is deaf develop specific forms of nonverbal communication, such as eye contact and touch [6,7]. Babies are also able to communicate from birth, and crying is an expression of their feelings of pain, hunger, fear, anger, and uncomfortable temperature (i.e., when they are too hot or too cold) [8].

Non-nutritive suckling habits are common behaviors in the 1^st^ year of life and can persist throughout childhood. These habits occur in both families with hearing impairment or have normal hearing [9]. Pacifier sucking is the most prevalent non-nutritive habit among preschool children [9]. This habit can interfere with the harmonious development of the face and dental arches, promote malocclusion, interfere with swallowing and phonation, and discourage breastfeeding [10-12]. In contrast, breastfeeding can prevent the establishment of non-nutritive sucking habits [12,13]. Up to now, little has been known about causes or pathways that lead to prolonged pacifier use, especially when comparing mothers with hearing impairment and those without. Understanding these pathways is important for planning future strategies for preventing pacifier use. Therefore, this study aimed to develop a pathway analysis for pacifier use among children whose mothers are hearing impairment or have normal hearing.

## 2. Materials and Methods

### 2.1. Study design, sampling, and research ethics committee approval

This cross-sectional study included 116 pairs of mothers and their children (2-5 years old) from Belo Horizonte, Brazil. This study consisted of two groups to be compared: Group 1 (G1) that included 29 pairs of mothers with hearing impairment and their children and Group 2 (G2) that included 87 pairs of normal-hearing mothers and their children. Data were collected from December 2017 to October 2018. The study was approved by the Research Ethics Committee of the Federal University of Minas Gerais (protocol 49803115.4.0000.5149). Mothers who agreed to participate signed a term of free and informed consent.

Mothers with hearing impairment were recruited from a referral center for individuals with hearing impairment in the city of Belo Horizonte. This center had 250 registered adolescents and adults with hearing impairment, of whom 40% were female. We invited all mothers with hearing impairment and their children to take part in the study. Normal-hearing mothers were recruited from two public day-care centers in Belo Horizonte.

### 2.2. Pilot study

In the pilot study, these methods were tested with 10 mothers with hearing impairment and 10 normal-hearing mothers of children enrolled at public schools. The pilot study showed that the methods and questionnaires proposed for the main study did not need to be changed.

### 2.3. Sample-size calculation

Due to the specificity of the sample, an estimation of the sample size needed for the main study was based on pacifier-sucking prevalence among children evaluated in the pilot study. For this purpose, we used online software from the Power Sample website (http://powerandsamplesize.com/). In the pilot study, the prevalence of the pacifier-sucking habit among children of mothers with hearing impairment was 40%, and the prevalence among children of normal-hearing mothers was 80%. We considered a 95% confidence level and 95% power. To achieve these values, the final sample was estimated to be 26 mothers with hearing impairment and 78 normal-hearing mothers (the participant ratio was 1:3, with one mother with hearing impairment to three normal-hearing mothers). To compensate for possible losses, 10% was added, and the final sample size consisted of 29 mothers with hearing impairment and 87 normal-hearing mothers.

### 2.4. Eligibility criteria

Deafness was confirmed by audiometry examinations of the mothers with hearing impairment at the time of the study to verify the type and degree of hearing loss [14]. Audiometry assessment was performed by one speech-therapy specialist, who confirmed the diagnosis of deafness.

We included mothers who had children aged from 2 to 5 years old and who were deaf with severe bilateral hearing loss; and mothers who had normal hearing. We excluded mothers with hearing impairment who had with cochlear implants, mothers with hearing impairment who were unaware of the Brazilian Sign Language (BSL), illiterate normal-hearing mothers, mothers with children with compromised systemic health conditions, physical and/or mental disabilities, or developmental/behavioral disorders (the children’s health problems were reported by the parents).

### 2.5. Data collection

Data were collected by means of mothers’ self-reports obtained using a structured questionnaire. The interviews were performed by trained researchers and administered in an identical manner to mothers with hearing impairment or had normal hearing. The speech therapist specialized in BSL, collected data from deaf mothers (RFN). A deaf mother with higher education, literate in BSL, previously trained the speech therapist (RFN) was asked to adapt the dental terms used in the interview. This mother was not included in the data collection. We contacted normal-hearing mothers through day-care centers. After a telephone call, the mothers and children were visited at their homes. The mothers’ data were collected by answering the self-reporting questionnaires. Similarly, we contacted the mothers with hearing impairment through the referral center. First, we contacted mothers with hearing impairment either by WhatsApp or cellphone messaging. We then visited the mothers with hearing impairment and their children at their homes. Mothers with hearing impairment answered the self-reporting questionnaires. The primary researcher performed data collection with the aid of a research assistant who was a specialist in both speech therapy and BSL.

### 2.6. Questionnaire

The following variables were collected by means of the questionnaires completed by mothers.

#### 2.6.1. Demographic factors

The demographic variables were child’s sex (female and male) and age of child and mother stated in years.

#### 2.6.2. Socioeconomic classification

A mother’s level of schooling was assessed by the number of years of schooling. Based on the median value (11.0; standard deviation [SD]=2.31), educational levels were categorized as either <11 years of schooling or ≥11 years of schooling.

Household income was categorized in terms of the Brazilian monthly minimum wage (BMMW), which corresponded to US$235.00 for all economically active members of the family at the time of data collection. Based on the median, the family BMMW income was categorized as either ≤2 BMMW or >2 BMMW.

As regards the variable of employment, the mother informed whether she worked outside the home or not.

#### 2.6.3. Pacifier-sucking habit duration

The habit of pacifier sucking beyond the age of 2 years reduces the likelihood of spontaneous correction of malocclusion [15]. Therefore, the length-of-time variable was dichotomized in children who had a pacifier-sucking habit for <24 months and those who had the habit for ≥24 months.

#### 2.6.4. Breast-feeding

The mother was asked whether the child had received exclusive breastfeeding (yes/no) and if she/he was using a bottle (yes/no).

#### 2.6.5. Childbirth variables

The mother was asked whether she had maternity leave and whether the child was born prematurely (yes/no).

#### 2.6.6. Assessment of anxiety in mothers

All mothers self-reported anxiety symptoms using the Brazilian version of the Beck Anxiety Inventory (BAI) [16]. Mothers with hearing impairment answered the BAI adapted to the BSL [17].

The BAI used a self-reported scale that aimed to assess the intensity of anxiety symptoms, differentiating the emotional symptoms from physical symptoms. This tool consisted of 21 items, such as “unable to relax, unsteady, nervous, afraid, heart pounding, fear of losing control, and indigestion,” among others. The response options ranged from 0 (absolutely not) to 3 (severe). The overall score could range from 0 to a had a severe level of anxiety [16,17].

### 2.7. Statistical analysis

The data were descriptively analyzed as either the frequency or the mean and SD for categorical and continuous variables, respectively. To verify any associations with pacifier use, we performed a pathway analysis using only the variables observed. The theoretical framework for this was based on a previous study [13]. This type of analysis allows the direct and mediating relationships between predictors and the duration of pacifier use to be assessed.

**Figure 1:**
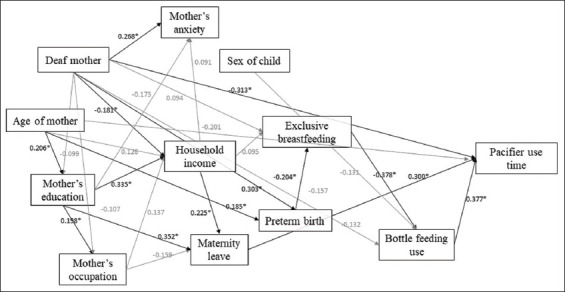
Pathway analysis showing the direct effects and standardized coefficients. *P<0.05.

The pathway analysis was performed using Mplus version 6.11 software. The maximum likelihood estimator was used because the analyses contained continuous variables, and the maximum likelihood estimator performed corrections for possible lack-of-normality concerns. A goodness-of-fit model was also evaluated using the standardized coefficient (SC) and global-fit indices. The SC values can be negative or positive, indicating the relational direction between variables. Values for an acceptable global fit were the root mean square error of approximation value <0.07, the standardized root mean square residual value <0.08, and Comparative Fit Index and Tucker-Lewis Index values >0.95 [18]. We ran an initial model and then performed a step-by-step removal of non-significant paths with *P*-values >0.40 for the final model. We considered the statistical significance when *P*-values were lower than 0.05. We kept the other associations in the model for goodness-of-fit reasons (Figure 1).

## 3. Results

The referral center for individuals with hearing impairment had 100 women registered, 34 of whom met the eligibility criteria. Five mothers with hearing impairment (14.7%) declined to participate. Of the 129 normal-hearing mothers contacted at the public day-care centers, 42 (32%) refused to receive home visits, and 87 (68%) agreed to participate in the study.

Table 1 presents the sample characteristics. The final sample included 116 pairs of mothers and children of both sexes (51.7% male and 48.3% female). The mean age of the mothers was 32 years (SD=7.4) and the mean age of the children was 3.15 years (SD=1.04). The majority of mothers had been to school for <11 years (81.9%), and their families had a monthly income of <US$900 (62.6%). The majority of mothers reported that their children were bottle-fed (69.8%) and had a pacifier-sucking habit (52.6%). Furthermore, 92.2% of mothers breastfed, but 65.5% reported that they did not exclusively breastfeed during the first 6 months after birth.

**Table 1:** Distribution of the population characteristics.

Variables	Frequency (%)
Groups of mothers	
Hearing impairment	29 (25.0)
Normal hearing	87 (75.0)
Mother’s level of schooling	
<11 years	95 (81.9)
≥11 years	21 (18.1)
Mother’s age (years)	
Mean (SD)	31.5 (±07.4)
Median (Min-Max)	32.0 (19-49)
Work outside the home	
No	35 (30.2)
Yes	81 (69.8)
Family income	
≤2 BMMW	67 (62.6)
>2 BMMW	40 (37.4)
Mother’s anxiety	
Mean (SD)	13.61 (±11.34)
Median (Min-Max)	11.0 (0-50)
Sex of child	
Male	60 (51.7)
Female	56 (48.3)
Child’s age (years)	
Mean (SD)	03.15 (±01.04)
Median (Min-Max)	03.0 (02-05)
Maternity leave	
Yes	77 (72.0)
No	30 (28.0)
Preterm birth	
Yes	12 (11.2)
No	95 (88.8)
Exclusive breastfeeding	
Yes	40 (34.5)
No	76 (65.5)
Child was the bottle-fed	
Yes	81 (69.8)
No	35 (30.2)
Pacifier use	
Yes	61 (52.6)
No	55 (47.4)

SD: Standard deviation; Min: Minimum; Max: Maximum

Table 2 and Figure 1 illustrate the pathway analysis. The duration of pacifier use was associated with bottle-feeding (*SC*=0.377, *P*=0.004), with children of normal-hearing mothers (*SC*=−0.313, *P*=0.005) and with mothers who had maternity leave (*SC*=0.300, *P*=0.004).

**Table 2:** Standardized estimated effects of results in structural models.

Pathways	Initial model	*P*-value	Model final	*P*-value
Pacifier duration ON				
Sex of child	0.077	0.477	-	-
Mother with hearing impairment	−0.318	0.009	−0.313	0.005
Age of mother	−0.211	0.064	−0.201	0.077
Maternity leave	0.309	0.003	0.300	0.004
Bottle-feeding use	0.375	0.004	0.377	0.004
Mother’s anxiety	0.035	0.770	-	-
Bottle-feeding use ON				
Exclusive breastfeeding	−0.378	0.000	−0.378	0.000
Sex of child	−0.131	0.117	−0.131	0.117
Mother who is deaf	0.132	0.115	0.132	0.115
Exclusive breastfeeding ON				
Household income	−0.098	0.294	−0.095	0.298
Preterm birth	−0.208	0.026	−0.204	0.021
Mother with hearing impairment	0.014	0.886	-	-
Mother’s occupation	0.096	0.301	0.094	0.305
Mother’s anxiety ON				
Mother who is deaf	0.268	0.002	0.268	0.002
Household income	0.091	0.353	0.091	0.353
Mother’s education	−0.173	0.071	−0.173	0.071
Preterm birth ON				
Mother with hearing impairment	0.303	0.000	0.303	0.000
Age of mother	0.185	0.031	0.185	0.031
Maternity leave ON				
Mothers works	−0.159	0.070	−0.159	0.070
Household income	0.225	0.014	0.225	0.014
Mother’s education	0.352	0.000	0.352	0.000
Household income ON				
Age of mother	0.126	0.151	0.126	0.151
Mother with hearing impairment	−0.181	0.032	−0.181	0.032
Mother’s education	0.335	0.000	0.335	0.000
Mothers works	0.137	0.115	0.137	0.115
Mother’s employment ON				
Mother’s education	0.198	0.026	0.198	0.026
Mother with hearing impairment	−0.107	0.234	−0.107	0.234
Mother’s education ON				
Age of mother	0.206	0.023	0.206	0.023
Mother with hearing impairment	−0.099	0.282	−0.099	0.282
Model fit				
RMSEA	0.025 (0.000-0.068#)		0.017 (0.000-0.064#)	
CFI	0.970		0.986	
TLI	0.952		0.977	
SRMR	0.059		0.059	

ON: Outcome on predictor; RMSEA; Root mean square error of approximation; CFI: Comparative fit index; TLI: Tucker-Lewis index; SRMR: Standardized root means square residual.

# Confidence interval 90%

Bottle-feeding was associated with the absence of exclusive breastfeeding (*SC*=−0.378, *P* < 0.001), and these factors were associated with premature birth (*SC*=−0.204, *P*=0.021). Premature birth was more prevalent among the children of mothers with hearing impairment (*SC*=0.303, *P*=0.000). Mothers with hearing impairment were also associated with higher anxiety scores (*SC*=0.268, *P*=0.002). Duration of pacifier use was lower among the children of the mothers with hearing impairment (SC=-0.313, *P*=0.005; Table 2).

Maternity leave was associated with higher family income (*SC*=0.225, *P*=0.014) and a higher level of education (*SC*=0.352, *P*=0.000). Lower family income, in turn, was associated with mothers with hearing impairment (*SC*=−0.181, *P*=0.032). Older mothers had a higher level of education (*SC*=0.206, *P*=0.023). Moreover, mothers who did work outside the home had a higher level of education (*SC*=0.198, *P*=0.026).

## 4. Discussion

The children of normal-hearing mothers used pacifiers for a longer period of duration than the children of mothers with hearing impairment. Normal-hearing mothers may have provided pacifiers more frequently to stop the discomfort of hearing their babies cry and for reassurance. A baby’s crying can cause exhaustion and fatigue in parents, which may ultimately lead to marital conflict [19]. Since a mother with hearing impairment does not hear her baby cry, she has no apparent reason for providing a pacifier. Mothers with hearing impairment may originally have introduced a pacifier due to cultural influences, but over time they probably stop providing it simply because they do not hear the child cry [6,7]. This would contribute to eliminating the habit in a shorter time. The children of mothers who had maternity leave also used pacifiers for longer periods. Maternity leave allows the mother to spend more time with the child without having to work [4]. This longer time may have led to these mothers offering a pacifier more frequently, contributing to the prolongation of the habit.

Mothers with hearing impairment had a higher rate of premature deliveries than normal-hearing mothers. A study conducted in the US found similar results [20]. A national sample of hospitalized patients was evaluated between 2008 and 2011 to compare birth outcomes in women with and without hearing loss. Women with hearing impairment were significantly more likely to deliver prematurely and have low birth-weight children compared with women without hearing loss [20]. Mothers with hearing impairment attended fewer prenatal appointments and reported receiving less information from medical staff than normal-hearing mothers. This may account for the increased number of premature deliveries [5] because adequate prenatal care can prevent risk factors for premature-delivery and prepare the mother for normal delivery [21]. Prenatal-care counseling is important for pregnant women and can encourage healthy behaviors and reduce the risk factors for problems during pregnancy [22,23]. Therefore, a lack of communication between healthcare staff and deaf mothers during prenatal care may have influenced premature deliveries among mothers with hearing impairment of this study.

We found that mothers with hearing impairment were more anxious than normal-hearing mothers. An American study with 1704 adults with hearing impairment and 3287 normal-hearing adults found that adults with hearing impairment had a higher reported rate of depression or anxiety disorder compared to normal-hearing individuals [24]. People with hearing impairment may have problems communicating with others, which could cause isolation and feelings of loneliness, capable of increasing anxiety [2]. Mothers with hearing impairment could also be anxious because they cannot hear their children cry [2]. Premature birth also increases mothers’ anxieties [25]. Often, a premature child needs special in-hospital care, such as the use of incubators and intubation devices. Preterm children may have difficulty with breastfeeding due to immature coordinated sucking, breathing, and swallowing [13]. Consequently, they may be more vulnerable to the use of pacifiers and bottle-feeding [13,26]. In the present study, premature children did not receive exclusively breastfed for 6 months. The above difficulties with breastfeeding among premature children may have influenced the decision to implement exclusive breastfeeding among the children in the present study. Moreover, limited communication with health professionals could lead to mothers with hearing impairment having difficulties in caring for their premature babies. All these factors would be capable of generating anxiety in mothers with hearing impairment [2].

The duration of pacifier use was associated with bottle-feeding and the use of a bottle was associated with the absence of exclusive breastfeeding [13]. A study carried out in Brazil with 250 children (2-5 years old) also found an association between the use of a bottle and pacifier use [13]. The World Health Organization has recommended that bottle-feeding and pacifiers should not be used by breastfeeding children [27]. Bottle-fed children may also find it more difficult to obtain milk from a breast due to “nipple confusion,” caused by differences in sucking techniques used for the bottle and breast, which can lead to weaning [28,29]. In turn, early weaning and bottle replacement could enhance the use of pacifiers [12].

Lower family income was associated with mothers with hearing impairment, and other studies have confirmed associations between low income and the community of individuals with hearing impairment [30-32]. Limited communication skills can limit access to the labor market for those with hearing impairment and can decrease job opportunities and job progression, factors related to the low family income. In the present study, lower family income was associated with the mothers’ lower levels of education. Similarly, older mothers had higher schooling levels, and they tended to work outside of the home. A lower level of schooling is correlated with a lower level of employment, and thus, with salary. In contrast, increased age was related to more years of study and more career experience. Consequently, these women had more job opportunities [33].

This is the first study of a pacifier pathway analysis between mothers with hearing impairment and normal-hearing mothers. It provided important insight into the children’s oral habits and how hearing loss could be related to these factors. The findings of this study led to the reflection that the mother’s understanding of her children’s crying had a strong influence on the duration of use of the pacifier. Although the number of mothers with hearing impairment in the study was small due to the low prevalence of the deaf population [1], it met the power analysis requirements for adequate sample size and this was not a limitation of the study. Future quantitative and qualitative studies with individuals with hearing impairment should be encouraged. Healthcare professionals should receive training to enable them to communicate with mothers with hearing impairment and help them learn to deal with their anxieties. Health professionals should advise mothers concerning the consequences of pacifier use for children and the best communication between professionals and families should be encouraged. This should be further investigated in future studies.

## 5. Conclusions

We concluded that children of normal-hearing mothers used pacifiers for longer periods of duration than the children of mothers with hearing impairment. The time of pacifier use was also influenced by bottle-feeding and maternity leave. In addition, premature children and high levels of anxiety were observed more frequently among mothers with hearing impairment.

These findings provided important information about the cultural pathway of pacifier use taken by families that had members with and without hearing impairment. It was noteworthy that the mother was shown to be an important caregiver regarding the health of her children. The effect of limited communication between health professionals and patients during prenatal care could affect the health of both mothers and their children. Future studies should be encouraged, and public policies should be implemented with a focus on the inclusion of mothers with hearing impairment. This study provided a new perspective on prolonged pacifier use in children of mothers with normal hearing in comparison with mothers with hearing impairment.
